# Comparison of video-based and presentation-based self-directed instruction for learning basic neuroangiography

**DOI:** 10.1186/s12909-026-10418-0

**Published:** 2026-10-03

**Authors:** Franka Stolze, Manuel Lehm, Daniel Behme, Erelle Fuchs, Roland Schwab

**Affiliations:** 1 University Hospital Magdeburg, University Clinic for Neuroradiology, Magdeburg, Germany; 2 Klinikum Region Hannover, Institute for Radiology, Hanover, Germany; 3 Otto von Guericke University Magdeburg, Research Campus STIMULATE, Magdeburg, Germany; 4 Department of Neuroradiology, Städtisches Klinikum Karlsruhe, Moltkestraße 90, Karlsruhe, 76133 Germany

**Keywords:** Angiography, Education, Navigation, Neurointervention, Medical education

## Abstract

**Background:**

Digital tools and simulation-based learning are playing an increasingly important role in medical education. Earlier studies have shown that video and virtual reality can be just as effective for teaching clinical skills as presentation-based teaching methods. This exploratory study compared two standardized self-directed instructional formats, video-based and presentation-based instruction, for teaching basic neuroangiographic skills. In addition to comparing objective and subjective learning outcomes between instructional formats, the study explored whether prior angiography experience influenced performance and moderated the effect of the teaching method.

**Methods:**

Twenty participants with different levels of prior angiography experience were randomly assigned to either a video-based group (VG; *n* = 9) or a presentation-based group (PG; *n* = 11). The VG watched expert-narrated recordings of a physician performing neuroangiography on a silicone flow model. The PG independently worked through static simplified slides derived from the same instructional material, including schematic illustrations and brief written annotations. Afterwards, all participants performed neuroangiography on the silicone flow model using three different catheter types (Vertebralis, Simmons 2, Headhunter). Overall performance was assessed using objective criteria (e.g., total and fluoroscopy time) and self-reported data based on learning and confidence.

**Results:**

For the Vertebralis catheter, those in the presentation group used significantly less fluoroscopy time, with the PG using 237.00 s on average, while the VG needed 369.00 s (*p* = 0.031). Nevertheless, participants rated the video method as significantly more helpful overall (*p* = 0.022). Moreover, for inexperienced participants video-based training led to more successful catheterization of the left vertebral artery (*p* = 0.048) and fewer path deviations (*p* = 0.048) when compared to the presentation-based approach.

**Conclusions:**

Video-based instruction may be a valuable adjunct to early neuroangiography training, especially for beginners. While it was associated with longer fluoroscopy times, participants perceived it as more helpful and showed improvements in certain procedural aspects. These findings highlight the potential benefits of integrating video-based elements into a blended learning curriculum for neuroangiography, and suggest that further optimization of instructional design is needed in medical education.

**Supplementary Information:**

The online version contains supplementary material available at https://doi.org/10.1186/s12909-026-10418-0.

## Introduction

In order to facilitate sufficient training in neurointerventional techniques, the development of highly specialised procedural skills is required. In current neurointerventional training, instruction usually combines expert explanation, demonstration, feedback, and supervised hands-on practice. Simulation-based learning has gained traction in recent years, being regarded as an effective method for educating medical students and residents, while also fostering motivation for continued learning in this field [1]. This is further supported by additional studies, demonstrating significant improvements in residents’ performance with simulation-based approaches [2]. It is furthermore highlighted that using simulation in neurointerventional training provides a low-risk environment, enabling trainees to develop procedural skills [3].

Although simulation-based training has been proven effective when teaching neuroangiography, it may not always be available. In this instance, video-based teaching presents a viable alternative in medical education demonstrating effectiveness in teaching fundamental skills. In this study, video-based teaching refers to a standardized expert-narrated recording that demonstrates the procedural steps, whereas expert-led instruction in previous studies usually involved direct in-person teaching with the opportunity for clarification and feedback. When comparing video-based teaching with expert-led instructions, no significant differences in the performance were observed between both groups, despite a significant improvement in surgical skills [4]. This suggests that video-based training may be a sufficient alternative, although it does not appear to be superior to traditional teaching methods. Other studies have shown that participants significantly improved their performance and confidence in venipuncture when using a video-based teaching method [5] Moreover, despite lower initial scores, a video-based method led to better retention of knowledge in comparison to traditional lectures at a 1-month follow-up [6]. These findings emphasize the potential of video-based learning to improve practical skills.

Given the inconsistent effectiveness across various areas of medical education, further investigation is needed to assess the applicability of video-based teaching in specialized fields like neurointervention. Studies in cardiological angiography can provide relevant insights into effective training approaches, as this type of intervention demands similar procedural skills to neuroangiography. One study demonstrated a significant change in performance-based skill scores, where individuals training with virtual-reality simulation showed significant improvement, while the performance of those receiving lecture-based teaching declined [7]. For beginners, the combination of lectures and simulation led to significantly improved procedural skills compared to lecture-based education alone [8]. These findings emphasize the benefits of integrating modern training methods into early procedural training, particularly for beginners.

Despite the variable outcomes, students show a clear preference for video lectures over live lectures [9]. Therefore, further research into video-based learning methods is needed to evaluate the best approaches for knowledge acquisition and trainee engagement.

Concurrent with recent technological advances, virtual-reality-based education has been increasingly incorporated across various areas of medical education. Studies have shown that it leads to improved retention of theoretical knowledge, enhances practical skills, and strengthens confidence [10]. Although the applicability of the findings from other fields of medical education remains questionable, a modernization of teaching methods is necessary to ensure neurointerventional training is in alignment with current technological developments. In this context, video-based teaching offers a modern and cost-effective approach, providing an accessible yet effective alternative to traditional lectures and simulation [11–13].

This exploratory study aims to compare two standardized self-directed instructional formats, video-based and presentation-based instruction, for learning basic neuroangiographic skills in a simulated setting. A secondary aim was to examine whether prior angiography experience influenced performance and whether inexperienced and experienced learners responded differently to the two instructional formats. The results may help refine blended neurointerventional curricula by identifying which learner groups benefit most from video-based teaching materials.

## Methods and materials

### Study design

The participants included third- and fourth-year medical students, as well as neurointerventional residents. A total of 20 participants were enrolled and randomly assigned to either the video-based group (VG; *n* = 9) or the presentation-based group (PG; *n* = 11). Randomization was performed using a simple concealed envelope allocation procedure, and allocation was not known to participants and researchers prior to enrollment. The slight imbalance between groups resulted from the small sample size and the use of simple randomization.

No formal a priori sample size estimation was performed, as this was designed as an exploratory pilot study. Participant recruitment was based on feasibility and the availability of eligible medical students and residents during the study period.

**Fig. 1:**
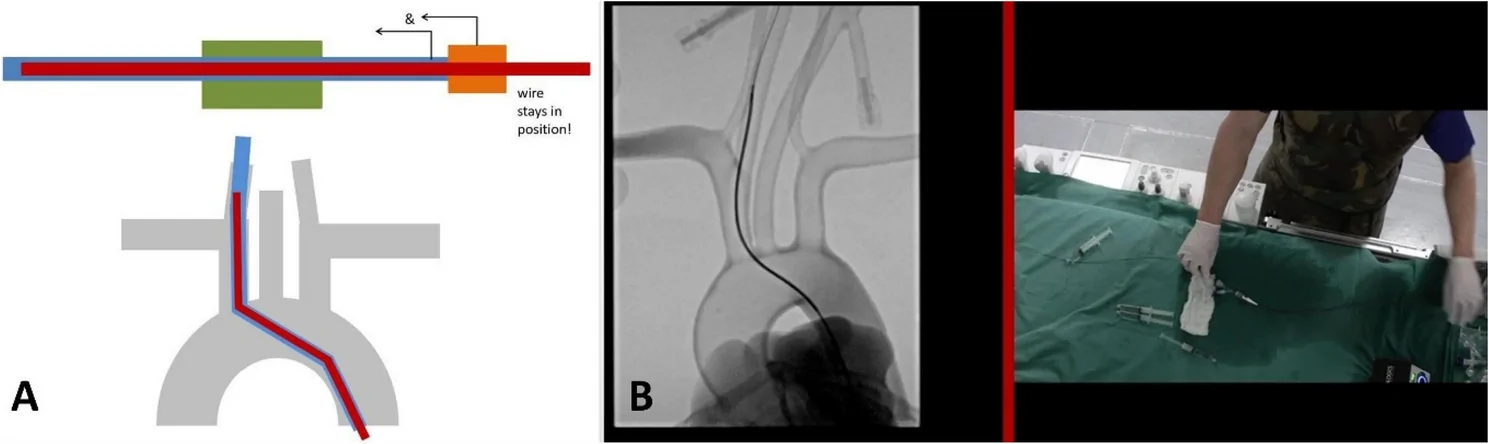
Exemplary comparison of the presentation (**A**) and the video (**B**) depicting the catheterization of the right carotid artery using the Simmons 2 catheter

Following randomization, the individual teaching sessions began. Participants in the video-based group (VG) watched three instructional videos in which a neurointerventional physician with over 8 years of experience demonstrated and explained the steps required to perform angiography of the right internal carotid artery (RICA), the left internal carotid artery (LICA) and the left vertebral artery (LV). Commonly employed 5 French selective catheters (Vertebral, Simmons 2, Headhunter) were used in a translucent silicone model, as illustrated in Fig. 1.

Simultaneously, participants in the presentation-based group (PG) viewed a static slideshow illustrating the same procedural steps in a simplified format (also demonstrated in Fig. 1). These participants received further explanations and were allowed to ask questions during the instructional part to further clarify the slideshow. This presentation was derived from the instructional videos and included schematic illustrations, along with brief annotations.

**Fig. 2:**
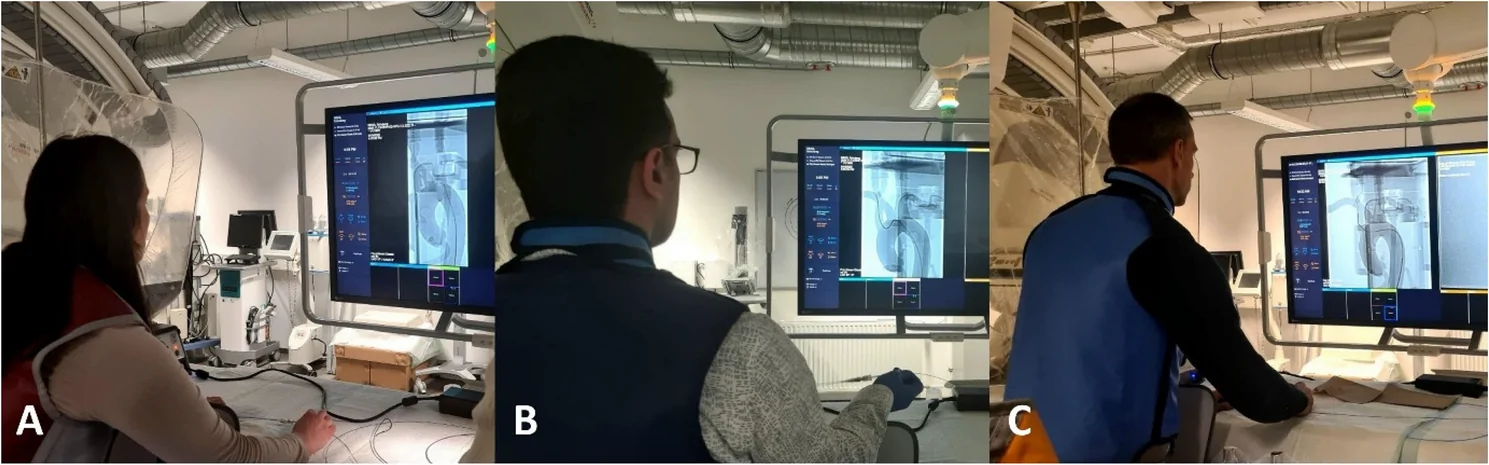
Participants performing a neuroangiography each using the Vertebralis catheter (**A**), the Simmons 2 catheter (**B**) and the Headhunter catheter (**C**)

After the instructional sessions, all participants independently performed the angiographic procedures on the same translucent silicone aortic arch flow model that had been was used for the demonstration material. An objective checklist, including all relevant arteries, was used for assessment. The angiography started with the selection of the appropriate catheter, starting with the Vertebralis catheter, followed by the Simmons 2 catheter and the Headhunter catheter. Thereafter, the target vessels were catheterized in the same order (see Fig. 2), as demonstrated in both the videos and the presentation. During the procedures, an observer completed an objective performance-based checklist in real time.

At the conclusion of the session, participants were encouraged to complete an anonymous self-evaluation questionnaire to collect further subjective data.

### Evaluation

The evaluation comprised an objective checklist and an anonymous self-evaluation questionnaire. In the objective checklist, total time used, fluoroscopy time, potential complications, path deviations, and other relevant criteria were assessed (see Suppl. Figure 1). Complications were defined as advancing the catheter without a wire, retracting it while leaving the tip of the wire outside of the image area, or failing to maintain visibility of the catheter or wire tip when progressing into vessels via fluoroscopic imaging.

The questionnaire recorded participants’ prior levels of experience. It also used a 5-point Likert scale to assess subjective success and perceived improvement in the subject. Lastly, participants could provide additional feedback through a free-text field. All participants completed the self-evaluation questionnaire (see Suppl. Figure 2).

### Statistical analysis

Upon completion of the evaluation, data were analyzed using IBM Statistical Package for Social Sciences (SPSS) version 28. Descriptive statistics were calculated for all objective and subjective variables.

Normality of the data was assessed using visual inspection of histograms. Given the small sample size and the use of non-parametric tests, no formal normality testing (e.g., Shapiro-Wilk) was conducted, as it was not considered to influence the chosen analytical approach.

VG and PG were compared for each catheter type using the Mann-Whitney-U-Test. The same tests were also applied to compare experienced and inexperienced participant performance overall. Participants were classified as experienced if they have independently performed at least one angiography prior to the study. To account for possible influence of experience-related effects, subgroup analyses were conducted for both the VG and PG using descriptive statistic and the Mann-Whitney-U-Test.

The study was designed as an explorative comparative analysis assessing multiple objective and subjective outcome parameters across learning modality and participants’ experience level. Due to the limited sample size (*n* = 20) and the number of interrelated endpoints, the analysis was considered exploratory rather than confirmatory. Accordingly, no formal correction for multiple comparisons was applied.

Results are reported as exact p-values and are interpreted in the context of effect sizes and consistency across related outcomes. Statistical significance was set at *p* < 0.05 for all tests.

## Results

### Objective parameters

A detailed breakdown of the objective parameters is shown in Table 1. The median fluoroscopy time was significantly lower in the PG (x͂ = 237.00 s) than in the VG (x͂ = 369.00 s) (*p* = 0.031, Table 1).

**Table 1:** Objective checklist parameters comparing the video-based group and presentation-based group for the Vertebralis catheter in all participants (n_total_ = 20; n_VG_ = 9; n_PG_ = 11)

criteria	group	x͂	IQR	Mann-Whitney-U Test *p*
correct tools used	VG	5	0	0.450
	PG	5	0	
correct preparation	VG	5	0.5	0.197
	PG	4	1	
catheter successful in the RICA	VG	4	2	0.101
	PG	5	1	
catheter successful in the LICA	VG	5	1	0.796
	PG	5	0	
catheter successful in the LV	VG	5	0	0.070
	PG	4	1	
total time used (min)	VG	7.00	6.00	0.117
	PG	6.00	3.00	
fluoroscopy time used (sec)	VG	369.00	382.00	**0.031**
	PG	237.00	152.00	
number of path deviations RICA	VG	3	4	0.129
	PG	0	1	
number of path deviations LICA	VG	0	2.5	1.000
	PG	0	1	
number of path deviations LV	VG	0	0	0.052
	PG	1	2	
number of complications	VG	0	1	1.000
	PG	0	1	
tip of the wire is always visible	VG	5	0	0.855
	PG	5	0	
successful handling	VG	4	1.5	0.105
	PG	4	1	
systematic approach	VG	4	1	0.362
	PG	5	1	
identifying and overcoming problems	VG	4	1	0.086
	PG	5	1	

*VG* video group, *PG* presentation group, *RICA* right internal carotid artery, *LICA* left internal carotid artery, *LV* left vertebral artery, *IQR* interquartile range

Bold type indicates statistically significant results (*p* < 0.05)

Prior angiography experience showed a more consistent association with objective performance than instructional format. Success rates were generally higher among experienced participants than among inexperienced participants, regardless of catheter type or learning method.

A comparative analysis of the objective parameters between experienced and inexperienced subgroups is outlined in Table 2. Experienced participants needed 35.41% less fluoroscopy time than inexperienced ones with the same catheter (U = 23.000, *p* = 0.043, *r* = -0.456, Table 2). They also demonstrated a significant superiority in handling the Vertebralis catheter (*p* = 0.019), a significantly better strategic approach (*p* = 0.017, Table 2), as well as better identification and resolution of problems (*p* = 0.007; *p* = 0.002, Table 2).

**Table 2:** Results of the objective checklist parameters of the inexperienced and experienced participants when using the Vertebralis catheter, the Simmons 2 catheter, and the Headhunter catheter respectively (n_total_ = 20; n_inexperienced_ = 10; n_experienced_ = 10)

criteria	group	Vertebralis catheter			Simmons 2 catheter			Headhunter catheter		
		x͂	IQR	Mann-Whitney-U Test p	x͂	IQR	Mann-Whitney- U Test p	x͂	IQR	Mann-Whitney-U Test p
correct tools used	Inexperienced	5	0	1.000	5	0.25	0.474	5	0.25	0.474
	Experienced	5	0		5	0		5	0	
correct preparation	Inexperienced	4	1	0.170	5	1	0.458	5	1	0.211
	Experienced	5	0.25		5	0		5	0	
catheter successful in the RICA	Inexperienced	4	2	0.052	3	1.25	**0.034**	5	1	0.582
	Experienced	5	0.25		5	1		5	0	
catheter successful in the LICA	Inexperienced	5	2	0.087	3	1.5	**0.001**	4	1.5	**0.011**
	Experienced	5	0		5	0.25		5	0.25	
catheter successfully placed in the LV	Inexperienced	5	1	1.000	3.5	2	0.141	5	1	0.582
	Experienced	5	1		5	1.25		5	0	
total time used (min)	Inexperienced	8.00	6.25	0.095	12.00	10.25	0.065	5.50	4.00	0.058
	Experienced	6.00	3.25		7.50	5.25		4.00	2.25	
fluoroscopy time used (sec)	Inexperienced	385.50	404.75	**0.043**	670.00	541.75	**0.029**	311.00	187.75	**0.012**
	Experienced	249.00	180.75		349.00	314.75		115.50	211.75	
number of path deviations RICA	Inexperienced	2	4	0.100	3	3.25	**0.022**	0.5	1.25	0.197
	Experienced	0	1.25		0.5	2.25		0	0.25	
number of path deviations LICA	Inexperienced	1	4	**0.034**	4.5	4.25	**0.003**	2.5	3.5	**0.006**
	Experienced	0	0		0	2		0	0.5	
number of path deviations LV	Inexperienced	0	2	1.000	3	2.5	**0.042**	0.5	1.25	0.308
	Experienced	0	1.25		0	1.25		0	0.25	
number of complications	Inexperienced	0	1	0.721	0	0	1.000	0	1.25	0.087
	Experienced	0	1		0	0		0	0	
tip of the wire is always visible	Inexperienced	5	0	0.211	5	0	1.000	5	0	1.000
	Experienced	5	1		5	0		5	0	
successful handling	Inexperienced	4	1	**0.019**	3	1.25	0.115	4	1.25	0.181
	Experienced	5	1		3	1.25		5	1	
systematic approach	Inexperienced	4	1	**0.017**	3	1	**0.045**	4.5	2	0.076
	Experienced	5	0		4	1.25		5	0	
identifying and overcoming problems	Inexperienced	3.5	1	**0.007**	3	1	0.095	4	1	0.248
	Experienced	5	1		4	1.25		5	1	

*RICA* right internal carotid artery, *LICA* left internal carotid artery, *LV* left vertebral artery, *IQR* interquartile range

Bold type indicates statistically significant results (*p* < 0.05)

Regarding the Simmons 2 catheter, similar patterns were found. Experienced participants had significantly higher success rates in catheterizing the RICA (*p* = 0.034) and the LICA (*p* = 0.001, Table 2). They had fewer path deviations for all blood vessels (RICA: *p* = 0.022, LICA: *p* = 0.003, LV: *p* = 0.042), and required 47.91% less fluoroscopy time (*p* = 0.029; Table 2). Further results are shown in Table 2.

Analysis of the results when using Headhunter catheter showed that experienced participants were significantly more successful in advancing into the LICA (*p* = 0.011), exhibited fewer path deviations on the way to the same blood vessel (*p* = 0.006), and required less fluoroscopy time than inexperienced participants (*p* = 0.012; Table 2). A complete overview of these results is provided in Table 2.

The analysis of inexperienced participants in the VG and the PG showed a significantly higher success rate of bringing the Vertebralis catheter into the LV when the video-based method was used compared to the presentation-based method (*p* = 0.048, Table 3). Accordingly, novices in the VG had fewer path deviations when advancing into the LV with this catheter (*p* = 0.048). These group differences are outlined in detail in Table 3.

**Table 3:** Objective checklist parameters comparing inexperienced participants in the video-based and presentation-based groups for the Vertebralis catheter (n_inexperienced_ = 10; n_inexperienced VG_ = 5; n_inexperienced PG_ = 5)

criteria	group	x͂	IQR	Mann-Whitney-U Test *p*
correct tools used	VG	5	0	1.000
	PG	5	0	
correct preparation	VG	5	1	0.524
	PG	4	0.5	
catheter successfully in the RICA	VG	3	1.5	0.071
	PG	5	1	
catheter successfully placed in the LICA	VG	5	2	1.000
	PG	5	1.5	
catheter successfully placed in the LV	VG	5	0	**0.048**
	PG	4	0.5	
total time used (min)	VG	12.00	12.5	0.095
	PG	6.00	5.5	
fluoroscopy time used (sec)	VG	637.00	594.50	0.056
	PG	247.00	242.00	
number of path deviations RICA	VG	4	3.5	0.103
	PG	0	2	
number of path deviations LICA	VG	1	4	1.000
	PG	1	2.5	
number of path deviations LV	VG	0	0	**0.048**
	PG	2	1.5	
number of complications	VG	0	1.5	1.000
	PG	0	1	
tip of the wire is always visible	VG	5	0	1.000
	PG	5	0	
successful handling	VG	3	1	0.119
	PG	4	0.5	
systematic approach	VG	4	0.5	1.000
	PG	4	1.5	
identifying and overcoming problems	VG	3	1.5	0.484
	PG	4	1.5	

*VG* video group, *PG* presentation group, *RICA* right internal carotid artery, *LICA* left internal carotid artery, *LV* left vertebral artery, *IQR* interquartile range

Bold type indicates statistically significant results (*p* < 0.05)

Inexperienced participants in the VG showed higher success rates (*r* = 0.655, *p* = 0.040, *n* = 10) and made fewer path deviations to advance into the LV (*r* = -0.640, *p* = 0.046, *n* = 10) than participants in the PG, similar to the results of inexperienced participants using the Headhunter catheter. However, there were no significant differences between the VG and the PG when only the results of experienced participants were analyzed (*p* > 0.050). Given the exploratory sample size and the absence of an a priori power calculation, these null findings are inconclusive and should not be interpreted as evidence of equivalence between the instructional formats.

### Subjective evaluation

In the self-evaluation questionnaire, participants of the video-based group rated the provided material as significantly more helpful than those using the presentation-based approach (U = 22.500, *p* = 0.022, *r* = -0.527). For a comprehensive summary of these results, see Table 4.

**Table 4:** Subjective evaluation comparing the video-based and presentation-based groups in all participants (n_total_ = 20; n_VG_ = 9; n_PG_ = 11) and in experienced participants only (n_experienced;_ = 10; n_experienced VG_ = 4; n_experienced PG_ = 6). Likert scales ranged from 1 to 5, with 1 indicating “strongly disagree” and 5 indicating “strongly agree.”

criteria	group	All			Experienced only		
		x͂	IQR	Mann-Whitney-U Test p	x͂	IQR	Mann-Whitney-U Test p
I have a better understanding of neuroangiography	VG	5	1	0.896	4	1.5	0.405
	PG	5	1		5	1.25	
I can explain the steps better	VG	4	1	0.395	4	2.25	0.552
	PG	5	1		4.5	1.25	
I know the differences between catheters	VG	5	0	0.591	5	0	1.000
	PG	5	1		5	0.25	
I could successfully apply my knowledge	VG	5	1	0.889	4.5	1.75	0.690
	PG	5	1		5	1	
I feel competent during performance	VG	4	2.5	0.259	4.5	1.75	0.714
	PG	4	1		4.5	1	
I can detect complications	VG	3	1.5	0.509	2.5	2.5	0.071
	PG	4	1		4	1.25	
The material was helpful	VG	5	0	**0.022**	5	0	0.076
	PG	4	1		4	1	
My understanding of this subject has deepened considerably.	VG	5	0.5	0.527	5	3	0.786
	PG	5	1		4.5	1	
The angiography simulation was close to reality	VG	4 (*n* = 7)	2	0.478	4	1.5	0.381
	PG	5 (*n* = 7)	1		4.5	1	
Participating has motivated me for further education	VG	5	1	0.276	5	0.75	1.000
	PG	5	0		5	0.25	
Teaching and simulation should be included in neuroradiological teaching	VG	5	0	0.450	5	0	1.000
	PG	5	0		5	0	
I will use the gained knowledge long-term	VG	5	1.5	0.543	5	0.75	1.000
	PG	5	1		5	0.25	

*VG* video group, *PG* presentation group, *IQR* interquartile range

Bold type indicates statistically significant results (*p* < 0.05)

There were no significant differences between the subjective evaluation of the VG and the PG when only the experienced participants were analyzed (Table 4). Given the small subgroup size, these findings are inconclusive rather than evidence of equivalent perceptions.

Amongst inexperienced participants, there were no significant differences in the overall evaluation of material usefulness, comprehensibility, or practical application between the VG and PG (*p* > 0.050).

Further analysis of the self-evaluation survey did not provide significant results. Nevertheless, all participants, regardless of the learning method or experience level, completely agreed that simulated angiography and its theoretical teaching should be integrated into the teaching methods of neuroradiology (x͂= 5 amongst all groups, Table 4).

Regarding the evaluation of long-term retention, inexperienced participants rated their retention at x͂ = 4, with a wider IQR in the VG (2.0) compared to the PG (1.0), whereas experienced participants in both groups gave a median rating of 5, with slightly greater variability in the VG compared to the PG (Table 4). Beyond long-term retention, inexperienced participants in the VG perceived the material as more helpful (x͂ = 5, IQR = 0.5) than those in the PG (x͂ = 4, IQR = 2.5, Table 4), although this difference was not significant.

Comprehensibility of the content was rated highly across both inexperienced groups, with participants in both the PG and VG reporting a median score of 5, though variation was slightly lower in the VG (Table 4). In addition, the successful application of the theoretically gained knowledge was evaluated higher by inexperienced participants in the VG (x͂ = 5, IQR = 1.0) compared to those in the PG (x͂ = 4, IQR = 2.5, Table 4). Inexperienced individuals reported substantial knowledge gains with both learning approaches (x͂ = 5 in both groups, Table 4).

Motivation to further engage with neuroangiography varied. While experienced participants rated their motivation similarly high in both groups (x͂ = 5), inexperienced participants in the PG reported a higher motivation level (x͂ = 5, IQR = 0.5) than those in the VG (x͂ = 4, IQR = 1.5), as is demonstrated in Table 4.

Three participants provided additional feedback of their opinion on the presentation. They suggested that “more emphasis should be placed on using rotation as a key technique for advancing the Simmons 2 catheter”. Furthermore, they recommended providing “more explanations about the differences between the catheters during the presentation”. Feedback indicated that the slides were partially unclear to some participants.

## Discussion

This exploratory simulation study compared video-based and presentation-based self-directed instruction for learning basic neuroangiographic skills. Across catheter types, prior angiography experience showed the most consistent association with performance, with experienced participants generally achieving higher success, fewer path deviations, and shorter fluoroscopy times. Instructional format showed a more selective pattern, without demonstrating a general superiority of one instructional format over the other. Rather, the results suggest that instructional format and prior experience interact in a more nuanced way. While participants in the PG completed the Vertebralis catheter task with shorter fluoroscopy time, inexperienced participants in the VG showed better technical precision for selected procedural aspects, including fewer path deviations and higher success in catheterizing the LV.

These findings indicate that video-based instructions help beginners develop a more accurate procedural and spatial model that potentially enhances their understanding for advancing catheters through vessels. This interpretation is consistent with multimedia learning theory, which suggests that combining visual demonstration with verbal explanation can support the integration of procedural information, particularly when learners have limited prior knowledge [14, 15].

However, the cost of this improved accuracy was a significant increase in fluoroscopy times, as video-trained novices generally required longer to complete tasks. This contrasts with studies on VR-based learning in cardiological angiography, which have shown improvements in both speed and precision [7, 16]. This discrepancy may stem from the passive nature of video-based teaching. An instructional video lacks the aspect of interactivity and does not provide immediate feedback, therefore possibly limiting the effectiveness of this approach. Interestingly, among experienced participants, the teaching method had minimal impact, suggesting that prior knowledge and hands-on experience reduced the dependence on the instructional format. This is in line with theories about professional development, which suggest that experienced participants may be less responsive to different instructional approaches once they have reached a certain skill-level in their performance [17].

No significant differences were observed between VG and PG with respect to the Simmons 2 and Headhunter catheters. Instead, experience level played a more important role, with experts significantly outperforming novices in speed, accuracy, and success rate across all catheters. This aligns with literature emphasizing deliberate practice as a key factor in mastering procedural skills [18]. Notably, novice learners in the VG demonstrated a slight advantage when using the Headhunter catheter, suggesting that learning with videos may be particularly beneficial for navigating more complex anatomy.

Subjectively, participants rated the video-based instructions as more helpful than the presentation-based teaching. This is especially true for those with prior experience in neuroangiography. Current trends in medical education are in alignment with this evaluation. They demonstrate that video formats are preferred for their clarity, realism, and the option to be watched again multiple times [9, 19].

However, experienced learners reported lower confidence levels in recognizing complications after video-based teaching. One possible reason for this could be the fact that the videos only focused on ideal scenarios and omitted error management, which is an important limitation of passive learning formats. Being able to identify errors and handle unexpected events is essential in neuroangiography, and these skills may require a more interactive approach than video-based teaching alone.

Among novice participants, both methods were well-received, with no significant differences in perceived usefulness. Encouragingly, self-reported knowledge improvement and anticipated retention were high in both groups. This finding is consistent with results of other studies that suggest that video-based learning can enhance long-term memory [20, 21]. However, follow-up studies are needed to verify the long-term impact of this learning method. Interestingly, novices who learned via presentations reported higher motivation to continue training. This could be attributed to the less guided format of a simplified presentation, which highlighted their existing knowledge gaps, thereby sparking curiosity and a desire to learn more about the procedure.

Overall, the participants of both groups agreed unanimously on the value of combining theoretical instruction with hands-on simulation. This is strongly supported by the growing consensus in medical education that procedural competence requires active practice, not just passive watching [22, 23].

These findings indicate that video-based learning can be a valuable addition to early-stage neuroangiography training. Expert-narrated videos can aid in building a fundamental understanding by providing a detailed visual procedural guide, as well as concise explanations of the procedure. This is consistent with previous research demonstrating that videos help learners understand both the conceptual comprehension and procedural execution of technical tasks [24, 25].

Video-based instruction alone, however, seems to be insufficient. The absence of real-time interaction or exposure to complications limits opportunities for learners to refine clinical skills like problem-solving, error management and decision-making, as is shown by the results where participants of the VG evaluated that they can detect complications less confidently than participants of the PG. These abilities can be developed when using interactive case-based learning [26]. Thus, blended learning approaches, combining video with hands-on training and live discussions, can lead to superior educational outcomes [27]. Therefore, we propose that an effective neuroangiography curriculum incorporates the combination of an instructional expert video (pre-session video), followed by case-based learning, interactive discussions, and simulation lab practice (utilizing silicone flow models or VR).

This structure aligns with the principles of cognitive load theory and Mayer’s cognitive theory of multimedia learning [28, 29]. Expert-narrated videos may reduce cognitive load by providing a coherent visual sequence and verbal explanation, thus reducing complexity by showing what learners can expect, followed by building skills through practice and feedback [28]. In fact, combining videos with high-fidelity simulations has already shown promising results in at least one procedural specialty [30]. Video-trained novices may therefore have formed a more detailed spatial and procedural model of catheter navigation. During initial hands-on performance, however, applying a newly constructed model requires deliberate step-by-step matching of fluoroscopic anatomy and catheter movement to the observed sequence. This real-time comparison can consume working-memory resources and time. Thus, the observed pattern, significantly longer fluoroscopy time in the overall VG and, among novices, numerically longer fluoroscopy alongside better LV success and fewer path deviations, may represent an early phase of skill acquisition in which precision develops before fluency. With repeated guided practice, increasing automation may reduce this time cost. In experienced learners, prior procedural schemes may reduce the added value of such instructional formats, which could explain why no significant differences between VG and PG were observed in this subgroup.

Video’s scalability, easy standardization, and usefulness in settings with limited resources provide major advantages when compared to presentation-based methods [31, 32]. When thoughtfully integrated into simulation, videos can support student preparation and engagement with materials in advance, with students reporting that videos help their preparation and understanding for simulation classes [33, 34]. This could optimize the productivity of hands-on sessions especially for beginners.

Several limitations must be considered. First the statistical power of this study is limited by the small sample size of 20 participants. This limitation also increases the risk of type 2 errors. Consequently, potentially relevant differences between the VG and PG may not have been detected. As a result, the findings of this study should be interpreted with caution and require confirmation in larger powered studies.

Second, simple randomization without stratification led to slightly unequal group sizes and may not have fully balanced prior angiography experience between groups. Third, multiple outcomes and catheter-specific comparisons were analyzed without formal correction for multiple testing. Consequently, significant p values should be interpreted cautiously. Fourth, the comparison was not between video-based teaching and a conventional live lecture, but between two self-directed instructional formats: an expert-narrated video and a static annotated slide presentation. Finally, the study assessed immediate performance in a silicone flow model and did not evaluate long-term retention or transfer to clinical angiography.

While this study supports the incorporation of video instruction into neuroangiographical procedural training, further research is needed to evaluate how well these skills can be transferred into clinical practice. Specifically, this present study only evaluated the usefulness of video instruction in the setting of aortic arch vessel selection. Subsequent studies should therefore examine whether these findings can be transferred to a broader range of angiographic procedures. Long-term follow-up studies could provide valuable insights into the efficiency of skill retention and on-the-job performance over time. Future studies should also explore more interactive formats, such as e-learning with embedded quizzes and videos or augmented reality applications. As interactive modalities that offer real-time feedback have previously demonstrated improvements in immediate learning, therefore possibly being able to combine the benefits of passive and active learning methods [35, 36].

## Conclusion

Video-based instruction may be an effective, scalable, and affordable method for teaching basic neuroangiographic skills, particularly for inexperienced learners. However, the present exploratory findings do not support replacing traditional hands-on or expert-led training with video-based instruction alone. Prior angiography experience appeared to be a major determinant of performance, and longer fluoroscopy times after video-based instruction highlight the need for explicit radiation-awareness training. Integrating expert-narrated videos into a blended curriculum with simulation, feedback, and discussion may be the most appropriate educational approach [37]. 

## Supplementary Information

Supplementary Material 1.

## Data Availability

All data generated or analysed during this study are included in this published article and its supplementary information.

## Abbreviations

VG: Video-based group
PG: Presentation-based group
LV: Left vertebral artery
LICA: Left internal carotid artery
RICA: Right internal carotid artery
IQR: Interquartile range
VR: Virtual reality

## References

[1] Kreiser K, Gehling K, Zimmer C. Simulation in der Angiografie – Erfahrungen aus 5 Jahren Lehre, Ausbildung und Forschung. Rofo. 2019;191(6):547–52. 10.1055/a-0759-2248. 30754054

[2] Spiotta AM, Rasmussen PA, Masaryk TJ, Benzel EC, Schlenk R. Simulated diagnostic cerebral angiography in neurosurgical training: a pilot program. J neurointerventional Surg. 2013;5(4):376–81. 10.1136/neurintsurg-2012-010319. 22576472

[3] Mirza S, Athreya S. Review of Simulation Training in Interventional Radiology. Acad Radiol. 2018;25(4):529–39. 10.1016/j.acra.2017.10.009. 29221857

[4] Nousiainen M, Brydges R, Backstein D, Dubrowski A. Comparison of expert instruction and computer-based video training in teaching fundamental surgical skills to medical students. Surgery. 2008;143(4):539–44. 10.1016/j.surg.2007.10.022. 18374052

[5] Pan M, Harcharik S, Moskalenko M, Luber A, Bernardo S, Levitt J. Instructional video for teaching venepuncture. Clin Teach. 2014;11(6):436–41. 10.1111/tct.12198. 25212929

[6] Sahu S, John J. To play or pause: Video-based or conventional lectures in medical classrooms. Indian J Physiol Pharmacol. 2021;65(1):55–9. 10.25259/IJPP_18_2020.

[7] Voelker W, Petri N, Tönissen C, et al. Does Simulation-Based Training Improve Procedural Skills of Beginners in Interventional Cardiology?>--A Stratified Randomized Study. J Interv Cardiol. 2016;29(1):75–82. 10.1111/joic.12257. 26671629

[8] Bagai A, O’Brien S, Al Lawati H, et al. Mentored simulation training improves procedural skills in cardiac catheterization: a randomized, controlled pilot study. Circ Cardiovasc Interv. 2012;5(5):672–9. 10.1161/CIRCINTERVENTIONS.112.970772. 23048053

[9] Khan M, Abdul Jabbar AB, Khan DA, Javed MA, Raja MHR, Muzaffar K. Medical students’ perception of live lectures compared to video lectures in basic sciences medical education: a cross-sectional survey of medical colleges in Pakistan. medRxiv. 2021:21249740.

[10] Sung H, Kim M, Park J, Shin N, Han Y. Effectiveness of Virtual Reality in Healthcare Education: Systematic Review and Meta-Analysis. Sustainability. 2024;16(19):8520. 10.3390/su16198520.

[11] Haerling KA, Miller CW. A Cost-Utility Analysis Comparing Traditional Clinical, Manikin-Based Simulation, and Screen-Based Virtual Simulation Activities. J Nurs Educ. 2024;63(2):79–85. 10.3928/01484834-20231205-04. 38316155

[12] Axelsson CGS, Healy MG, Wolbrink TA, King J, Sanders ES, Phitayakorn R. Applying a novel cost-evaluation framework to assess video-based neurosurgery education. Am J Surg. 2020;220(3):604–9. 10.1016/j.amjsurg.2020.01.018. 31982093

[13] Karkera S, Devendra N, Lakhani B, Manahan K, Geiser J. A review on modern teaching and learning techniques in medical education. JETM. 2024;2(1). 10.59652/jetm.v2i1.128.

[14] Augestad KM, Butt K, Ignjatovic D, Keller DS, Kiran R. Video-based coaching in surgical education: a systematic review and meta-analysis. Surg Endosc. 2020;34(2):521–35. 10.1007/s00464-019-07265-0. 31748927

[15] Clerkin R, Patton D, Moore Z, Nugent L, Avsar P, O’Connor T. What is the impact of video as a teaching method on achieving psychomotor skills in nursing? A systematic review and meta-analysis. Nurse Educ Today. 2022;111:105280. 10.1016/j.nedt.2022.105280. 35139443

[16] Jensen UJ, Jensen J, Ahlberg G, Tornvall P. Virtual reality training in coronary angiography and its transfer effect to real-life catheterisation lab. EuroIntervention: J EuroPCR Collab Working Group Interventional Cardiol Eur Soc Cardiol. 2016;11(13):1503–10. 10.4244/EIJY15M06_05. 26547247

[17] Ericsson KA. Deliberate practice and the acquisition and maintenance of expert performance in medicine and related domains. Acad Med. 2004;79(10 Suppl):70–81. 10.1097/00001888-200410001-00022 15383395

[18] McGaghie WC, Issenberg SB, Cohen ER, Barsuk JH, Wayne DB. Does simulation-based medical education with deliberate practice yield better results than traditional clinical education? A meta-analytic comparative review of the evidence. Acad medicine: J Association Am Med Colleges. 2011;86(6):706–11. 10.1097/ACM.0b013e318217e119. PMC3102783 21512370

[19] Perrin M, Schäfer M, Bart P-A, Hahnloser D. Advancing pre-clinical surgical education by using intuitive short videos. BMC Med Educ. 2025;25(1):323. 10.1186/s12909-025-06895-4. PMC11869550 40022066

[20] Ludwig S, Schuelper N, Brown J, Anders S, Raupach T. How can we teach medical students to choose wisely? A randomised controlled cross-over study of video- versus text-based case scenarios. BMC Med. 2018;16(1):107. 10.1186/s12916-018-1090-y. PMC6034339 29976211

[21] Roy H, Andurkar PP, Doibale SK, Kharatmol MA, Waghmare SS, Suresh J. Effect of video lectures in comparison with text reading on memory retention among medical students. A randomised controlled trial, Aurangabad, Maharashtra, India. IJAR. 2022;10(11):1088–92. 10.21474/IJAR01/15769.

[22] Issenberg SB, McGaghie WC, Petrusa ER, Lee Gordon D, Scalese RJ. Features and uses of high-fidelity medical simulations that lead to effective learning: a BEME systematic review. Med Teach. 2005;27(1):10–28. 10.1080/01421590500046924. 16147767

[23] Cook DA, Hamstra SJ, Brydges R, et al. Comparative effectiveness of instructional design features in simulation-based education: systematic review and meta-analysis. Med Teach. 2013;35(1):e867–98. 10.3109/0142159X.2012.714886. 22938677

[24] Hurtubise L, Martin B, Gilliland A, Mahan J. To play or not to play: leveraging video in medical education. J Graduate Med Educ. 2013;5(1):13–8. 10.4300/JGME-05-01-32. PMC3613297 24404220

[25] Srinivasa K, Chen Y, Henning MA. The role of online videos in teaching procedural skills to post-graduate medical learners: A systematic narrative review. Med Teach. 2020;42(6):689–97. 10.1080/0142159X.2020.1733507. 32174211

[26] Yoo M-S, Park J-H. Effect of case-based learning on the development of graduate nurses’ problem-solving ability. Nurse Educ Today. 2014;34(1):47–51. 10.1016/j.nedt.2013.02.014. 23518068

[27] Li M, Hong Y, Wu A, et al. The effectiveness of blended learning in nursing and medical education: An umbrella review. Nurse Educ Pract. 2025;86:104421. 10.1016/j.nepr.2025.104421. 40483931

[28] van Merriënboer JJG, Sweller J. Cognitive load theory in health professional education: design principles and strategies. Med Educ. 2010;44(1):85–93. 10.1111/j.1365-2923.2009.03498.x. 20078759

[29] Mayer RE. The past, present, and future of the cognitive theory of multimedia learning. Educational Psychol Rev. 2024;36(1):8. 10.1007/s10648-023-09842-1.

[30] Drummond D, Arnaud C, Thouvenin G, et al. An innovative pedagogic course combining video and simulation to teach medical students about pediatric cardiopulmonary arrest: a prospective controlled study. Eur J Pediatr. 2016;175(6):767–74. 10.1007/s00431-016-2702-1. 26847430

[31] Mora Carpio AL, Romero-Reveron R, Vasquez M, et al. Leveraging technology to improve access to medical education in anatomy and physiology in resource-limited settings: A mixed methods study. Anat Sci Educ. 2024;17(6):1283–98. 10.1002/ase.2474. 38954744

[32] Leone TA, Rich W, Finer NN, Educational Perspectives. Neoreviews. 2006;7(12):e597–601. 10.1542/neo.7-12-e597.

[33] Botelho MG. Evaluation of student use of videos to support learning in a simulation laboratory course: A perception and analytics approach. J Invest Clin Dent. 2019;10(4):e12453. 10.1111/jicd.12453. 31560416

[34] Fukuta J, Morgan J. First-person perspective video to enhance simulation. Clin Teach. 2018;15(3):231–5. 10.1111/tct.12659. 28493407

[35] Subramanian A, Timberlake M, Mittakanti H, Lara M, Brandt ML. Novel educational approach for medical students: improved retention rates using interactive medical software compared with traditional lecture-based format. J Surg Educ. 2012;69(4):449–52. 10.1016/j.jsurg.2012.05.013. 22677580

[36] Dengri C, Gill A, Chopra J, et al. A Review of the Quiz, as a New Dimension in Medical Education. Cureus. 2021;13(10):e18854. 10.7759/cureus.18854. PMC8597672 34804707

[37] Mao BP, Teichroeb ML, Lee T, Wong G, Pang T, Pleass H. Is Online Video-Based Education an Effective Method to Teach Basic Surgical Skills to Students and Surgical Trainees? A Systematic Review and Meta-analysis. J Surg Educ. 2022;79(6):1536–45. 10.1016/j.jsurg.2022.07.016. PMC9356715 35933308

