# Supplementary material for: Comparison of video-based and presentation-based self-directed instruction for learning basic neuroangiography
Source: BMC Med Educ. 2026 Oct 3;26:1572. doi: 10.1186/s12909-026-10418-0 (PMC13633628; doi:10.1186/s12909-026-10418-0)
Supplement: Supplementary file 1 [file 12909_2026_10418_MOESM1_ESM.docx]

Online supplementary data

**Supplementary Table 1** Objective assessment checklist used to evaluate participants’ performance during neuroangiography.

RICA, right internal carotid artery; LICA, left internal carotid artery; LV, left vertebral artery

Supplementary Table 2 Self-assessment questionnaire completed by participants after the practical session.
